# Mesenchymal Stem Cells as Promoters, Enhancers, and Playmakers of the Translational Regenerative Medicine

**DOI:** 10.1155/2017/3292810

**Published:** 2017-06-22

**Authors:** Andrea Ballini, Salvatore Scacco, Dario Coletti, Stefano Pluchino, Marco Tatullo

**Affiliations:** ^1^Department of Basic Medical Sciences, Neurosciences and Sense Organs, University of Bari “Aldo Moro”, Bari, Italy; ^2^Institut de Biologie-Biology of Adaptation and Aging (B2A), Université Pierre et Marie Curie Paris 6, Paris, France; ^3^Department of Clinical Neurosciences-Division of Stem Cell Neurobiology, Wellcome Trust-Medical Research Council Stem Cell Institute and NIHR Biomedical Research Centre, University of Cambridge, Cambridge, UK; ^4^Biomedical Section, Tecnologica Research Institute, Crotone, Italy

Since their first isolation and characterization by Friedenstein et al. in 1974 [1], mesenchymal stem cells (MSCs) were proven essential for tissue regeneration and homeostasis.

Over the years, thanks to a better understanding of the molecular mechanisms underlying the therapeutic effects of MSCs, several approaches with MSC-based therapies have been proposed, in order to treat different human diseases [2].

In this light, MSCs are currently being tested in preclinical in vivo settings as well as in early-stage clinical trials for their ability to modulate immune responses, fostering wound healing and tissue regeneration of various tissue types and organs, including the skin, bone, cartilage, brain, muscle, and tendons [3].

As defined by the International Society for Cellular Therapy (ISCT) in 2006, culture-expanded MSCs [4], harvested from bone marrow, adipose tissue, umbilical cord, dental tissues, and other sources, are being studied in clinical trials across evidence-based practice and numerous regulatory jurisdictions worldwide [5–9].

Recent reports have demonstrated therapeutic effects by MSCs on animal models, explained by the ability of MSCs to be activated by signals from injured tissues: in these damaged areas, MSCs showed regenerative behaviour, with the promotion of the tissue healing, and paracrine activities, with the secretion of anti-inflammatory factors [10].

Experimental in vivo models showed that the direct administration of the same factors secreted by MSCs replicated the local anti-inflammatory effects, highlighting the double role of MSCs as players of tissue regeneration and as regulators of the local environment in many pathological conditions [10–12].

Actually, several clinical trials focused on the role of MSCs in the treatment of the autoimmunity are currently in progress, with the aim to verify new therapeutic approaches for the most common and severe diseases, such as Crohn's disease, type 1 diabetes, and systemic lupus erythematosus (see www.clinicaltrials.gov) [10, 11].

Despite the numerous barriers to their clinical use, MSCs have shown sufficient promise to garner a primary place in the field of regenerative medicine. MSC-based cell therapies have significant implications for human health: clinical studies are greatly needed to confirm or to stimulate the basic and translational researches aimed to reach cutting-edge results.

The key deliverable for the scientific community involved in developing MSC clinical applications could be to better understand the mechanisms underlying the MSC commitment and behaviour, by creation of MSC-based platforms which could provide a useful guidance on selection of proper clinical trial subjects based on hypothesis-driven mechanistic predictors of response [13].

Although all are such promising results, there are still severe limitations that currently delay the implementation of stem cell models in practice assays. The articles published in this special issue make use of MSC application for translational regenerative medicine and try to address some of these limitations. The original research articles as well as the review articles collected in this special issue will support the research community in approaching the goal of obtaining models for the lab that can better mimic the in vivo conditions.

Various methodologies have been employed to improve the cell phenotype generated from MSCs, for example, by modulation of biochemical activity, creation of scaffolds, and addition of growth factors to the culture medium. Furthermore, several topics regarding molecular mechanisms controlling MSC differentiation are covered.

Significant findings have been made in clinical research on application of MSCs for translational regenerative medicine: the reviews and original articles in this special issue highlight new methodological paradigms that challenge current thinking in clinical research and boost the development of clinical-grade MSCs under good manufacturing practice protocols and conditions, an essential step towards clinical applications.

The special issue has reported articles on MSCs used as therapeutic aid in clinical and surgical applications. The topics mainly reported have been the MSC therapy for spinal cord injuries (A. Zadroga et al. and F. Salamanna et al.) and the cell therapy as promising aid for retinal injuries due to ischemic damage (L. Li et al.), as well as for the proper management of stroke (C. Tan et al.).

The most reported translational use of MSC therapy is related to bone tissue regeneration: in fact, many authors have investigated about the osteogenic ability of different stem cell types, with several scaffolds as support to regeneration (Y. Kim et al.), as well as about the use of MSCs to treat osteonecrosis in the maxillofacial region (T. Lombard et al.); the dental-derived MSCs used for bone tissue engineering seem to be still the most used by the S.I. authors (F. Posa et al.), even if bone marrow cells have been used both in bone regeneration (A. Scarano et al. and D. C. Bonfim et al.) and also in the therapy of acute liver diseases (C. O. Kieling et al.).

A special attention has been paid also to umbilical cord stem cells, due to their large application on translational medicine (I. Arutyunyan et al.) and due to their ability to produce exosomes with interesting abilities (B. Zhang et al.).

Some authors have focused their researches on the influence of growth factors and proteins of ECM on MSCs (A. Youssef et al. and M. Moslem et al.) as well as the ability by MSCs to release GFs during their specific stage of life (C. G. Pfeifer et al.) or in combination with adipose-derived stem cells in bone regenerative protocols (F. Mussano et al.).

An interesting general view has been also given on topics related to MSCs of general interest, topics regarding the innovative ways to manage and store the stem cells for therapeutic uses in most different tissues (A. Bissoyi et al. and R. Rohban and T. R. Pieber).

Finally, some authors have also reported interesting aspects on regulatory and quality requirements in MSC research (P. Galvez-Martin et al.).

In this special issue, the editors together with the involved authors have well described the MSCs in their different but fundamental roles as promoters, enhancers, and playmakers of the translational regenerative medicine. Starting from the contents of our issue, the scientific community will be stimulated to experiment new ideas, to improve the knowledge of the MSCs, and to speed up their clinical application, so to improve the future therapies.

*Andrea Ballini* *Andrea Ballini* *Salvatore Scacco* *Salvatore Scacco* *Dario Coletti* *Dario Coletti* *Stefano Pluchino* *Stefano Pluchino* *Marco Tatullo* *Marco Tatullo*

## References

[1] Friedenstein A. J., Chailakhyan R. K., Latsinik N. V., Panasyuk A. F., Keiliss-Borok I. V. (1974). Stromal cells responsible for transferring the microenvironment of the hemopoietic tissues. Cloning in vitro and retransplantation in vivo. *Transplantation*.

[2] Parekkadan B., Milwid J. M. (2010). Mesenchymal stem cells as therapeutics. *Annual Review of Biomedical Engineering*.

[3] Lechanteur C., Briquet A., Giet O., Delloye O., Baudoux E., Beguin Y. (2016). Clinical-scale expansion of mesenchymal stromal cells: a large banking experience. *Journal of Translational Medicine*.

[4] Galipeau J., Krampera M., Barrett J. (2016). International Society for Cellular Therapy perspective on immune functional assays for mesenchymal stromal cells as potency release criterion for advanced phase clinical trials. *Cytotherapy*.

[5] Fazzina R., Iudicone P., Fioravanti D. (2016). Potency testing of mesenchymal stromal cell growth expanded in human platelet lysate from different human tissues. *Stem Cell Research & Therapy*.

[6] Squillaro T., Peluso G., Galderisi U. (2016). Clinical trials with mesenchymal stem cells: an update. *Cell Transplantation*.

[7] Marrelli M., Paduano F., Tatullo M. (2015). Human periapical cyst-mesenchymal stem cells differentiate into neuronal cells. *Journal of Dental Research*.

[8] Paduano F., Marrelli M., White L. J., Shakesheff K. M., Tatullo M. (2016). Odontogenic differentiation of human dental pulp stem cells on hydrogel scaffolds derived from decellularized bone extracellular matrix and collagen type I. *PloS One*.

[9] Di Benedetto A., Brunetti G., Posa F. (2015). Osteogenic differentiation of mesenchymal stem cells from dental bud: role of integrins and cadherins. *Stem Cell Research*.

[10] Uccelli A., Prockop D. J. (2010). Why should mesenchymal stem cells (MSCs) cure autoimmune diseases?. *Current Opinion in Immunology*.

[11] Prockop D. J., Oh J. Y. (2012). Mesenchymal stem/stromal cells (MSCs): role as guardians of inflammation. *Molecular Therapy*.

[12] Shi Y., Hu G., Su J. (2010). Mesenchymal stem cells: a new strategy for immunosuppression and tissue repair. *Cell Research*.

[13] Boregowda S. V., Phinney D. G. (2016). Quantifiable metrics for predicting MSC therapeutic efficacy. *Journal of Stem Cell Research & Therapy*.

